# Celiac disease and risk of myasthenia gravis – nationwide population-based study

**DOI:** 10.1186/s12883-018-1035-2

**Published:** 2018-03-12

**Authors:** Sujata P. Thawani, Thomas H. Brannagan, Benjamin Lebwohl, Peter H. R. Green, Jonas F. Ludvigsson

**Affiliations:** 1 Department of Neurology, New York University School of Medicine, New York, NY USA; 2 Peripheral Neuropathy Center, Neurological Institute, Columbia University, College of Physicians and Surgeons, New York, NY USA; 3 Celiac Disease Center, Department of Medicine, Columbia University College of Physicians and Surgeons, New York, NY USA; 4 Department Medical Epidemiology and Biostatistics, Karolinska Institutet, 171 77 Stockholm, Sweden; 5 Department of Pediatrics, Örebro University Hospital, Örebro University, Örebro, Sweden; 6 Division of Epidemiology and Public Health, School of Medicine, University of Nottingham, Nottingham, UK

**Keywords:** Autoimmune, Celiac, Gluten, Myasthenia gravis

## Abstract

**Background:**

Case reports suggest there may be an association between celiac disease (CD) and myasthenia gravis (MG).

**Methods:**

We identified 29,086 individuals with CD in Sweden from 1969 to 2008. We compared these individuals with 144,480 matched controls. Hazard ratios (HRs) for future MG (identified through ICD codes) were estimated using Cox regression.

**Results:**

During 326,376 person-years of follow-up in CD patients, there were 7 MG cases (21/million person-years) compared to 22 MG cases in controls during 1,642,273 years of follow-up (14/million person-years) corresponding to a HR of 1.48 (95% CI = 0.64–3.41). HRs did not differ when stratifying for age, sex or calendar period. HRs were highest in the first year after follow-up, though insignificant. Individuals with CD were at no increased risk of MG more than 5 years after CD diagnosis (HR = 0.70; 95% CI = 0.16–3.09).

**Conclusion:**

This study found no increased risk of MG in patients with CD.

## Background

Celiac disease (CD) is an immune mediated chronic disease that affects the proximal small intestine [1]. An increased susceptibility to other immune mediated diseases such as type 1 diabetes (T1D), rheumatoid arthritis, and Sjogren’s disease has been well described in association with CD [2]. Myasthenia Gravis (MG) is a rare immune mediated disease involving the neuromuscular junction with a prevalence estimate of 11.71 to 32/100,000, which also includes cases of mild or pure ocular MG [3–6]. CD is common with a 1% prevalence estimate [7–9]. Reports of fatigue and muscle weakness may suggest that MG is also present in CD as described in several case reports [10–12]. CD has also been found to precede a documented diagnosis of MG in the population based Swedish Patient Register with a 1.7 odds ratio of developing MG after a diagnosis of CD, [13] This analysis did however utilize hospital-based CD diagnoses with the subsequent risk of overestimating the association between CD and MG. In Sweden, like in the United States, many patients with CD are never admitted to the hospital [14].

Serological markers associated with MG have been reported in CD cases [15]. A retrospective analysis of 23 acetylcholine receptor (AChR) antibody patients, revealed one patient with both IgA-endomysial and IgA tissue transglutaminase antibodies. This single patient had a subsequent endoscopic evaluation that was consistent with CD [15]. In another series, one out of 70 CD patients and one out of ten control patients with systemic lupus erythromatosus had elevated muscle AChR antibody levels [16]. In addition, human leukocyte antigen (HLA) types B8 and DR3 appear to be associated with both MG and CD suggesting a shared immune origin [17, 18].

The objective of our study was to examine the relative risk and absolute risk of developing MG in a nationwide population-based sample of patients with biopsy-verified CD.

## Methods

We used nationwide biopsy data from Sweden’s 28 pathology departments to identify CD cases. These data were then linked to the Swedish Patient Register containing both inpatient and hospital-based outpatient data to ascertain the risk of MG [19].

### Study participants

Between October 2006 and February 2008 we collected data from small intestinal biopsy reports from all 28 Swedish pathology departments. Biopsies had been conducted between 1969 and 2008 (Table 1). We obtained data on date of biopsy, topography (duodenum or jejunum), morphology codes consistent with villous atrophy (VA) which was used as our definition for CD) and personal identity number [20]. For each patient with CD, the government agency Statistics Sweden identified up to five reference individuals from the Total Population Register. Matching criteria were age, sex, calendar year and county. We then removed individuals with data irregularities, and thereafter had data on 29,096 individuals with CD and 144,520 reference individuals (identical to the cohort used in our earlier paper on CD and mortality) [21].

**Table 1:** Characteristics of participants presented as n (%)

Characteristics	Reference individuals	Celiac disease
Total	144,480	29,086
Age at study entry (years)		
0–19	58,851 (40.7)	11,802 (40.6)
20–39	26,378 (18.3)	5311 (18.3)
40–59	32,237 (22.3)	6471 (22.2)
60-	27,014 (18.7)	5502 (18.9)
Sex		
Female	89,523 (62.0)	17,999 (61.9)
Male	54,957 (28.0)	11,087 (38.1)
Calendar Period		
-1989	20,371 (14.1)	4104 (14.1)
1990–1999	59,860 (41.4)	12,055 (41.4)
2000-	64,249 (44.5)	12,947 (44.4)
Nordic	136,242 (94.3)	28,129 (96.7)
Type 1 diabetes	596 (0.4)	956 (3.3)
Autoimmune thyroid diseases	2502 (1.7)	1435 (4.9)

Autoimmune thyroid disease, for definitions

### Celiac disease

CD was defined as VA (Marsh stage 3) according to biopsy report [22]. Having a positive CD serologic test result was not a prerequisite for CD diagnosis although 88% of individuals in a random subset of individuals with VA and available data on serology had positive CD serology at the time of biopsy [23]. Additional details on the data collection has been published elsewhere [23]. On average, three tissue specimens were examined for each biopsy report.

### Myasthenia gravis (MG)

MG was defined according to relevant international classification of disease (ICD) codes in the Swedish National Patient Register: ICD7: 744.00; ICD8: 733.00; ICD9: 358A; ICD10: G70.0. The Patient Register started in 1964 and contains diagnoses from ICD-7 through ICD-10. The Patient Register included inpatient care but since 2001, hospital-based outpatient care has also been included. We did not have access to data on acetylcholine receptor antibody results, but National Patient Register-based MG diagnosis has been validated against the Stockholm MG Cohort. This analysis found that 147/177 diagnoses in the Patient Register could be confirmed against the Stockholm MG Cohort and the positive predictive value of MG is 83% [13].

Individuals with MG before CD diagnosis and corresponding controls based on dates were excluded from all prospective analyses (*n* = 8 and *n* = 22 respectively). We then excluded another 2 CD patients and 2 controls due to data irregularities (uncertainties regarding date of death) and another 18 reference individuals since their index individual with CD had been excluded and analyses were performed retaining strata. Hence, there remained 29,086 individuals with CD and 144,480 matched controls.

### Other covariates

Data on the following potential confounding factors were collected from the government agency *Statistics Sweden*: country of birth (Nordic vs. not Nordic), educational level (≤9 years of primary school, 2 years of high school, 3–4 years of high school, college/university) and socioeconomic status according to six categories (according to the European Socioeconomic Classification, ESeC: levels 1, 2, 3 + 6, 7, 8, and 9) [24]. Missing data on education and socioeconomic status (4 and 31% respectively) were fitted into separate categories for the statistics. We also identified individuals with T1D and autoimmune thyroid disease defined according to relevant ICD codes.

### Statistical analyses

We estimated hazard ratios (HR) for MG using Cox regression. We used an internal stratification system and each stratum (consisting of one CD patient and up to five controls) was analyzed separately and a summary HR was calculated. Through visual inspection of log-minus-log curves we found that the proportional hazard assumption was valid. The attributable risk percent (the proportion of MG in patients with CD that could be explained by the underlying CD) was estimated by 1–1/HR. Follow-up started at first biopsy with CD diagnosis and at the same time in matched controls. Follow-up ended when one of the following occurred: death, emigration, first MG diagnosis, or end of study (December 31, 2009).

In a priori analyses, we examined the time-dependent risk of MG (time since CD diagnosis: < 1 year, 1–4.99 years; and > 5 years). We also performed analyses stratified by age at diagnosis of CD (≤19, 20–39, 40–59 and ≥60 years), sex, and calendar period (− 1989, 1990–99, 2000-). In a separate analysis we adjusted for country of birth, education, socioeconomic status, T1D, and autoimmune thyroid disease.

In order to examine the temporal association between CD and MG, we also calculated the odds ratios (ORs) for having a diagnosis of MG prior to the first biopsy for CD. For this analysis, we used conditional logistic regression comparing each individual with CD with his or her matched control.

A post-hoc power analysis found that we had an 80% power at a significance level of 0.05 to detect a HR of 3.1 for MG.

We used SPSS 22 to calculate statistics. *P*-values < 0.05 were considered statistically significant.

### Ethics

This study was approved by the Regional Ethical Review Board in Stockholm (2006/633–31/4). Because this was a register-based study, none of the participants was contacted and all data were anonymized prior to data analysis [25].

## Results

### From our prior paper

The median age of CD diagnosis was 30 years (range 0–95 years). Median year of diagnosis and first biopsy was 1998 (range 1969–2008). Median age at first diagnosis with MG was 44 years in CD patients and 48 years in controls. Most study participants were women (Table 1), and were diagnosed in the 1990s or later.

### Risk of future MG in patients with CD

During 326,376 person-years of follow-up in CD patients, there were seven cases of MG (21 per million person-years). This compares with 22 MG cases in controls during 1,642,273 person-years of follow-up (14 per million person-years). This corresponded to a HR of 1.48 (95% CI = 0.64–3.41).

Adjusting for socioeconomic status, education, and country of birth, had a marginal influence on the HR (HR = 1.68; 95% CI = 0.71–3.94). Excluding the first year of follow-up, the HR decreased somewhat (HR = 1.29; 5% CI = 0.53–3.14; Table 2). The lower risk estimate beyond the first year signaled a higher risk in the immediate year after CD diagnosis, although the HR in the first year failed to attain statistical significance (HR = 8.09; 95% CI = 0.70–94.04; *p* = 0.095).

**Table 2:** Risk of myasthenia gravis in patients with celiac disease according to follow-up

Follow-up	HR; 95% CI	*P*-value	Observed vs. Expected	Person-years	Incidence rate^a^	Excess risk^a^	Attributable percentage
All	1.48; 0.64–3.41	0.356	7 vs 5	326,376	21	7	32
< 1 year	8.09; 0.70–94.04	0.095	1 vs 0	28,773	35	30	88
1–5 years	2.19 0.69–6.91	0.182	4 vs 2	108,012	37	20	54
> 5 years	0.70; 0.16–3.09	0.640	2 vs 3	189,591	10	−4	−42

Beyond 1 year of follow-up

^a^Myasthenia gravis cases per 1,000,000 person-years in patients with celiac disease

The difference in risk estimates between men and women was not statistically significant (Table 3)(p for interaction = 0.290).There was also no difference in risk estimates according to age groups (p for interaction = 0.629) or calendar period (p for interaction = 0.863).

**Table 3:** Risk of MG myasthenia gravis in patients with celiac disease. Stratified analyses

Follow-up	HR; 95% CI	*P*-value	Observed vs. Expected	Person-years	Person-years Incidence rate^a^	Excess risk^a^	Attributable percentage
Age (yrs)							
< 20	2.60; 0.50–13.60	0.257	2 vs 1	147,218	14	8	62
20–39	1.99; 0.40–9.92	0.402	2 vs 1	60,204	33	16	50
40–59	0.58; 0.07–4.58	0.602	1 vs 2	75,119	13	−10	−74
> =60	1.55; 0.33–7.37	0.581	2 vs 1	43,835	46	16	36
Sex							
Female	2.28; 0.82–6.37	0.166	5 vs 2	204,000	24	14	56
Male	0.79; 0.18–3.52	0.760	2 vs 3	122,376	16	−4	−26
Calendar period							
-1989	1.33; 0.27–6.48	0.726	1 vs 1	85,893	12	3	25
1990–99	1.16; 0.26–5.29	0.848	2 vs 2	161,814	12	2	14
2000-	2.10; 0.57–7.66	0.264	3 vs 1	78,669	38	20	52

^a^Myasthenia gravis cases per 1,000,000 person-years in patients with celiac disease

### Prior MG in patients with CD

Conditional logistic regression found no increased risk for earlier MG in CD (OR = 1.70; 95% CI = 0.79–3.66).

## Discussion

This nationwide population-based study found no association between CD and future MG. The high HR in the first year of follow-up (8.09) compared to the HR after more than 5 years (0.70) suggests surveillance bias just after MG diagnosis.

Recently, Fang et al. also using Swedish data, examined the autoimmune spectrum in MG and found an OR of 1.7 for earlier CD in MG [13]. The authors however ascertained CD using the Patient Register. Until the year 2000, the Patient Register only contained inpatient data. CD rarely requires hospital admission and is primarily managed and diagnosed in the outpatient setting [14, 26]. Therefore, this earlier study may have identified a larger proportion of cases with severe CD or severe comorbidities, thereby pushing the risk estimate upwards.

Previous case reports and retrospective analyses of the association between CD and MG have examined serological markers shared between patients with both CD and MG [12, 15, 16]. Although serological markers could not be specifically examined in this analysis, validation studies of CD diagnoses with serological studies was performed in a subset of CD patients undergoing small intestinal biopsy, yielding a positive predictive value of 95% [23]. The diagnosis of MG has also been previously validated in the Patient Register [13, 27]. In addition to its population-based setting, another strength of this analysis is that the CD diagnoses were based on small intestinal biopsy reports [23], which was the gold standard for diagnosis of CD in Sweden throughout the study period.

Although our study used nationwide population based data, this analysis lacked statistical power. MG is rare in the general population with prevalence estimates ranging from 15 to 179 cases per million [7, 8]. The diagnosis of MG was determined by diagnosis codes in this study, and should be corroborated by electrophysiological and serum studies. This information was not available in the Patient Register thus some cases may have been missed. Some data suggest that, among patients with CD, adherence to a gluten free diet is associated with protection against other autoimmune diseases [28, 29]. However, we did not have information on dietary adherence amongst participants diagnosed with CD to assess if this had any effect on a later diagnosis of MG.

## Conclusions

In conclusion, in this population-based cohort study we did not demonstrate an increased risk of MG in CD. This could partly be due to insufficient power since our power analysis suggested that we could only demonstrate a 3.1-fold increased risk of MG in CD. However, our study shows that the absolute risk of MG is low in CD and we therefore discourage routine testing for acetylcholine receptor (AChR) antibodies in CD.

## Abbreviations

CD: Celiac disease
CI: Confidence Interval
HR: Hazard ratio
ICD: International Classification of Disease (codes
MG: Myasthenia Gravis
OR: Odds ratio
VA: Villous atrophy
